# Prescribed medicine use and extent of off‐label use according to age in a nationwide sample of Australian children

**DOI:** 10.1111/ppe.12870

**Published:** 2022-02-16

**Authors:** Andrea L. Schaffer, Claudia Bruno, Nicholas A. Buckley, Rose Cairns, Melisa Litchfield, Simon Paget, Helga Zoega, Natasha Nassar, Sallie‐Anne Pearson

**Affiliations:** ^1^ Centre for Big Data Research in Health UNSW Sydney Sydney New South Wales Australia; ^2^ Biomedical Informatics and Digital Health The University of Sydney Sydney New South Wales Australia; ^3^ NSW Poisons Information Centre The Children’s Hospital at Westmead Sydney New South Wales Australia; ^4^ Faculty of Pharmacy The University of Sydney Sydney New South Wales Australia; ^5^ The Children’s Hospital at Westmead Clinical School The University of Sydney Sydney New South Wales Australia; ^6^ Centre of Public Health Sciences Faculty of Medicine University of Iceland Reykjavik Iceland; ^7^ Menzies Centre for Health Policy The University of Sydney Sydney New South Wales Australia

**Keywords:** attention deficit disorder with hyperactivity, Australia, gastroesophageal reflux, paediatrics, pharmacoepidemiology

## Abstract

**Background:**

Medicine prescribing for children is impacted by a lack of paediatric‐specific dosing, efficacy and safety data for many medicines.

**Objectives:**

To estimate the prevalence of medicine use among children and the rate of ‘off‐label’ prescribing according to age at dispensing.

**Methods:**

We used population‐wide primarily outpatient dispensing claims data for 15% of Australian children (0–17 years), 2013–2017 (*n* = 840,190). We estimated prescribed medicine use and ‘off‐label’ medicine use according to the child's age (<1 year, 1–5 years, 6–11 years, 12–17 years) defined as medicines without age‐appropriate dose recommendations in regulator‐approved product information. Within off‐label medicines, we also identified medicines with and without age‐specific dose recommendations in a national prescribing guide, the Australian Medicines Handbook Children's Dosing Companion (AMH CDC).

**Results:**

The overall dispensing rate was 2.0 dispensings per child per year. The medicines with the highest average yearly prevalence were systemic antibiotics (435.3 per 1000 children), greatest in children 1–5 years (546.9 per 1000). Other common medicine classes were systemic corticosteroids (92.7 per 1000), respiratory medicines (91.2 per 1000), acid‐suppressing medicines in children <1 year (47.2 per 1000), antidepressants in children 12–17 years (40.3 per 1000) and psychostimulants in children 6–11 years (27.0 per 1000). We identified 12.2% of dispensings as off‐label based on age, but 66.3% of these had age‐specific dosing recommendations in the AMH CDC. Among children <1 year, off‐label dispensings were commonly acid‐suppressing medicines (35.5%) and topical hydrocortisone (33.1%); in children 6–11 years, off‐label prescribing of clonidine (16.0%) and risperidone (13.1%) was common. Off‐label dispensings were more likely to be prescribed by a specialist (21.7%) than on‐label dispensings (7.5%).

**Conclusions:**

Prescribed medicine use is common in children, with off‐label dispensings for medicines without paediatric‐specific dosing guidelines concentrated in classes such as acid‐suppressing medicines and psychotropics. Our findings highlight a need for better evidence to support best‐practice prescribing.

**Synopsis**

**Study question**

What are the most common on‐ and off‐label medicines prescribed to children in Australia?

**What’s already known**

Due to a lack of paediatric‐specific dosing data for many medicines, off‐label prescribing in outpatient settings is common. Most recent studies focus on hospital settings, specific medicine classes, or small samples.

**What this study adds**

In this nationwide study of prescribing primarily in outpatient settings, we showed that prescribed medicine use is common in children, with 1 in 2 dispensings for medicines without paediatric‐specific dosing guidelines either in the official product information or a national prescribing guide, commonly acid‐suppressing medicines and psychotropic medicines. Our findings highlight a need for better evidence in this population to support best‐practice prescribing, minimise low‐value care and improve outcomes.

## 1 BACKGROUND

Prescribing medicines for children can be challenging as many are not licensed for use in this population due to limited or no evidence about efficacy and safety.^1^ Therefore, treating clinicians often prescribe medicines that lack paediatric‐specific dosing information in regulator‐approved product information (PI), commonly referred to as “off‐label” use. While the lack of dose recommendations in PI for children does not necessarily mean that medicines are ineffective in this population, medicine pharmacokinetics frequently differ in children and adults, meaning adult dosing regimens cannot be directly extrapolated to children.^2^ This may place children at greater risk of harm^3^ with studies finding that medicine‐related adverse events in children are more likely to involve off‐label or unlicenced medicine use.^4^

While estimates vary, off‐label prescribing is typically high in paediatric outpatient settings.^5^ One 2019 US study found that physicians prescribed at least one off‐label medicine at 19% of visits, typically for unapproved conditions, and was increasing over time.^6^ In France, 45% of children was prescribed an off‐label or unregistered medicine in general practice.^7^ A European study found that medicines commonly prescribed off‐label to children in general practice included topical and systemic steroids, and oral contraceptives,^8^ while in a US study, anti‐infectives, respiratory and nervous system medicines accounted for three quarters of community off‐label prescribing in children.^6^ However, most studies published in the past 5 years have focussed on inpatient settings,^9^, ^10^, ^11^ specific medicine classes^12^, ^13^, ^14^, ^15^, ^16^ or in small samples.^7^

While existing studies identify areas of potentially problematic prescribing, only a few recent studies have used contemporary, nationwide data to describe the extent of prescribed medicine use internationally^17^, ^18^ and none in Australian children. By understanding which medicines are commonly prescribed on‐ and off‐label, we can identify research targets to elucidate our understanding of safety, identify potential low‐value prescribing and improve quality of care. In this study, we used outpatient dispensing claims data from a representative, nationwide 15% sample of all Australian children to estimate the prevalence of prescribed medicine use by age group and the rate of ‘off‐label’ medicine use according to the child's age at dispensing.

## 2 METHODS

### 2.1 Study population selection and data source

We conducted a cross‐sectional descriptive study of medicine dispensing over 5 years (2013–2017). Australia maintains a publicly funded, universal healthcare system entitling citizens and eligible residents to subsidised medicines through the national Pharmaceutical Benefits Scheme (PBS). We used PBS dispensing claims for a 15% random sample of PBS‐eligible children aged 0–17 years between January 2013 and December 2017. These data capture all medicines listed on the PBS schedule dispensed in the community, private hospitals and on discharge from some public hospitals. This collection does not capture medicines prescribed to public hospital inpatients, private dispensings (i.e., for medicines not listed on the PBS or outside of the PBS‐approved indication) and over‐the‐counter medicines. The PBS schedule can be accessed on their website,^19^ while information on prescription and over‐the‐counter medicines available in Australia can be accessed via the Australian Register of Therapeutic Goods.^20^ The data include each child's month and year of birth, and we set the day of birth to the 15^th^ of the month for analyses.

### 2.2 Outcomes

We included all medicines except those used primarily to treat cancer (World Health Organisation (WHO) Anatomical Therapeutic Chemical (ATC) code L) as children with cancer are typically treated as inpatients where we do not have capture of dispensing data. For our primary analysis, we classified medicines according to the WHO ATC first (anatomical subgroup) and second (therapeutic subgroup) levels, and for secondary analyses, we used the third level (pharmacological subgroup).^21^ We defined age groups as infants (<1 year), toddler and preschool (1–5 years), early childhood (6–11 years) and adolescent (12–17 years) to reflect categories commonly used in Australia.^22^, ^23^ To allow for comparison with international studies, we have also replicated key analyses using the categories <2 years (infants and toddlers) and 2–5 years (preschool) instead, which are available in the Supplementary Files.

We next classified each medicine dispensing as on‐label or off‐label according to the child's age on the date of dispensing. Medicines were “on‐label” if there were age‐appropriate dose recommendations for at least one indication in regulator‐approved PI and “off‐label” if there were no age‐appropriate recommendations in the PI. Importantly, our classification relates only to the age of the child; we did not undertake analyses by indication or prescribed dose, as we did not have this information in our data. Our approach is consistent with other research.^5^, ^8^, ^24^

Within off‐label medicines, we also identified medicines where there were age‐appropriate dose recommendations in the Australian Medicines Handbook Children's Dosing Companion (AMH CDC).^25^ The AMH is an independent national formulary and prescribing guide and consolidates prescribing information on a wide range of medicines including all medicines registered by the Therapeutic Goods Administration on the Australian Register of Therapeutic Goods. The AMH CDC provides specific guidance on age‐appropriate doses in children, based on their age, weight and/or body surface area. It identifies when medicine use is off‐label and provides age‐appropriate recommended doses for use in children where it is deemed to be clinically appropriate and supported by evidence.

We considered a medicine's route of administration (e.g., oral and injection) as recommendations sometimes varied by formulation. For fixed‐dose combination products not specifically mentioned in the AMH CDC, we considered the recommendations for each individual component. For a small number of medicines (1.8% of all formulations), recommended doses were provided for those over a minimum weight rather than by age. As we did not have person‐level information on weight, we used the 97^th^ percentile of growth chart weights for each child's age and sex at the time of dispensing. For children ≤24 months, we used the WHO growth charts^26^ while for children >24 months, we used those from the Center for Disease Control^27^ consistent with Australian guidelines.^28^

### 2.3 Statistical analysis

We calculated the dispensing rate as the number of dispensings per child‐year according to age, sex and remoteness of area of residence (major city, inner regional, outer regional, remote and very remote) averaged over all years combined. We also calculated the average yearly prevalence of medicine use by WHO ATC categories as the number of children with at least one dispensing per 1000 children in each year averaged over all years. To calculate the number of children or child‐years for the denominator, we used mid‐year age‐specific populations from the Australian Bureau of Statistics.^29^ For the area of residence, we only had data on the population ≤19 years, and so, we interpolated the population ≤17 years based on Australia‐wide estimates. We adjusted population estimates for the 15% sampling frame. For prevalence according to ATC categories, we restricted reporting to medicine classes dispensed to ≥1 per 1000 child‐years.

Within each medicine class, we categorised the first dispensing from a prescription (original) by prescriber type, specialist or nonspecialist (general practitioner (GP), allied health practitioner or dentist) and calculated the proportion of new prescriptions that were by a specialist physician. To understand patterns of medicine dispensing (chronic or sporadic use), we also calculated the mean number of dispensings in the first year (365 days) after the first observed dispensing for each child in each class, excluding children with their first dispensing in the last year of follow‐up.

The analysis of off‐label use was at the dispensing level. To identify the medicines most commonly dispensed off‐label by age, we calculated the proportion as the number of dispensings considered off‐label by age divided by all dispensings in each age group. We reported off‐label dispensing per 1000 child‐years using ABS population estimates as described above, as well as the proportion of off‐label dispensings that were prescribed by a specialist physician. We used r Version 4.0.2 and sas Version 9.4 for all analyses.

### 2.4 Missing data

Month and year of birth was available for all children. Sex and remoteness were missing for 0.03% and 0.7% of children respectively; they were only excluded from analyses involving these variables but included in other analyses (e.g., by age).

### 2.5 Ethics approval

This study was approved by the New South Wales Population and Health Services Research Ethics Committee (no. 2013/11/494) with a waiver from seeking individual consent. Data access was granted by the Australian Government Services Australia External Request Evaluation Committee (no. MI7681).

## 3 RESULTS

Our study population included 840,190 children (49.3% female) with 8,219,772 dispensings (Table 1). Overall, the dispensing rate was 2.0 dispensings per child‐year and was lowest in children <1 year (1.6 dispensings) and highest in the 12‐ to 17‐year age groups (2.3 dispensings). The yearly dispensing rate was greatest in children in major cities and lowest in remote or very remote areas (Table 1). Overall dispensing rates using the age categories <2 years and 2–5 years (instead of <1 year and 1–5 years) are in Table S1 and show a similar pattern.

**TABLE 1:** Characteristics of study population, 2013–2017

	No. of children with ≥1 dispensings	Child‐years	Dispensings	Dispensings per child‐year
Total	840,190	4,026,231	8,219,722	2.0
Age, years				
<1	129,793	232,557	374,206	1.6
1–5	353,683	1,166,277	2,573,065	2.2
6–11	332,607	1,340,014	2,398,899	1.8
12–17	323,085	1,281,384	2,904,068	2.3
Sex				
Female	413,959	1,959,701	3,860,992	2.0
Male	425,949	2,066,530	4,357,246	2.1
Remoteness area^a^				
Major cities	598,497	2,730,618^b^	5,862,531	2.1
Inner regional	149,849	829,088	1,499,295	1.8
Outer regional	71,676	400,334	688,300	1.7
Remote or very remote	13,985	97,775	108,420	1.1

Note

Sex missing for *n* = 282; remoteness missing for *n* = 6183.

^a^ Remoteness area for each child's first dispensing.

^b^ Person‐years for remoteness area are approximate and may not add up to the total.

### 3.1 Prevalence of medicine use by age

**FIGURE 1:**
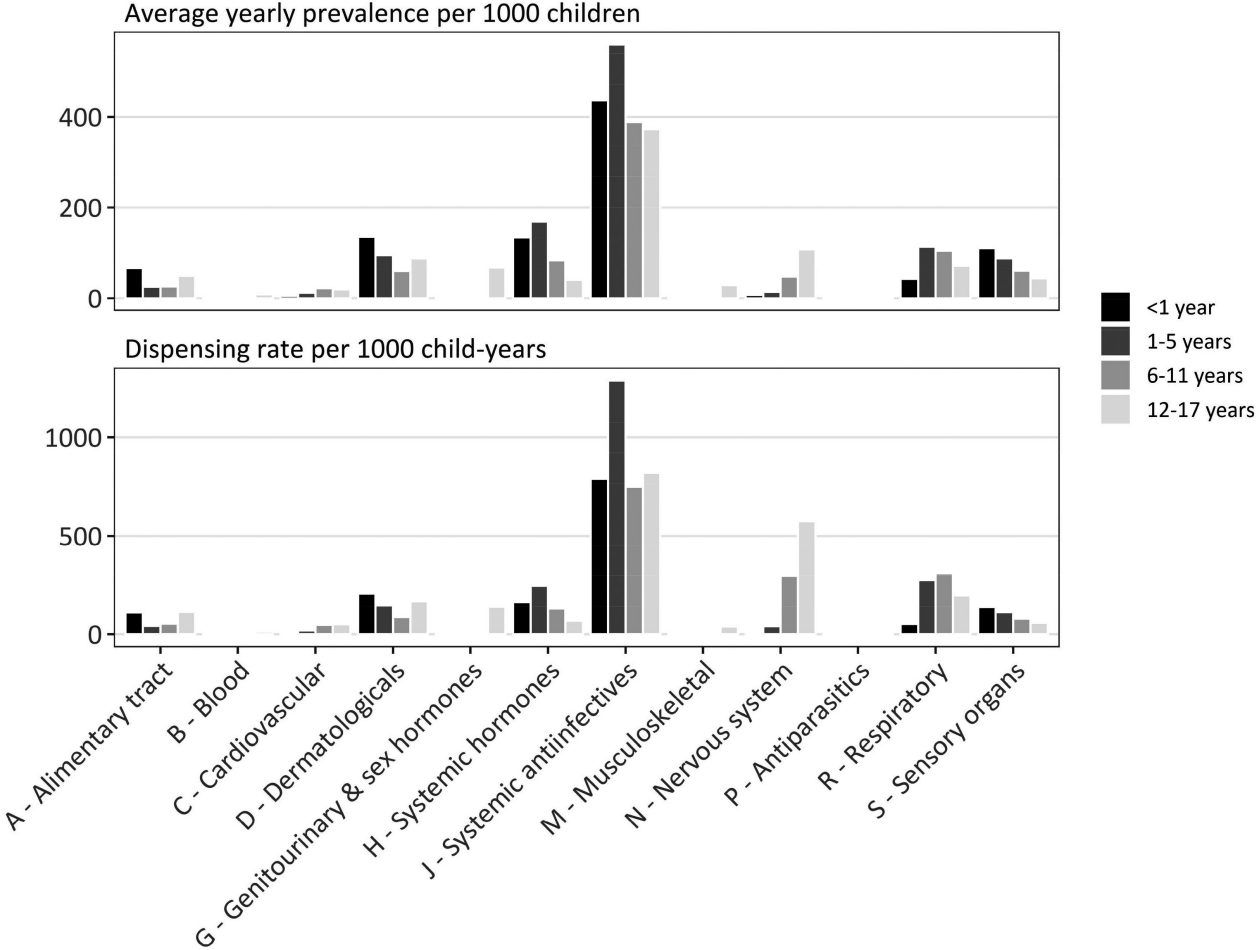
Medicine dispensing by age group. Average yearly prevalence of medicine use and dispensing rate by World Health Organisation Anatomical Therapeutic Chemical anatomical classification and age group, 2013–17

Systemic anti‐infectives were the most dispensed WHO ATC anatomical medicine class across all age groups (Figure 1) driven by broad‐spectrum penicillins and first‐generation cephalosporins (Table S2). The classes at the WHO ATC therapeutic subgroup level with the highest average yearly prevalence were antibacterials (435.3 per 1000 children), obstructive airway disease medicines (91.2 per 1000) and systemic corticosteroids (92.7 per 1000) (Table 2).

**TABLE 2:** Dispensing prevalence by WHO ATC therapeutic subgroup, restricted to subgroups with an average yearly prevalence ≥1 per 1000 children, 2013–17

Therapeutic subgroup	Most common examples of medicines in subgroup	Average yearly prevalence per 1000 children					Prescribed by specialist, %	Dispensings per child in first year of observed use^a^	
		<1	1–5	6–11	12–17	All (0–17)		Two or more, %	Mean
A—alimentary tract									
A01—stomatological preparations	nystatin, amphotericin B	13.0	2.8	0.9	1.2	2.3	2.2	15.2	1.2
A02—acid‐related disorders	omeprazole, esomeprazole	47.2	4.9	8.0	19.3	13.1	18.0	35.8	2.2
A03—functional gastrointestinal disorders	metoclopramide, domperidone	0.3	0.4	1.6	12.5	4.7	4.1	11.6	1.2
A04—antiemetics and antinauseants	ondansetron, prochlorperazine^b^	3.6	12.8	9.5	9.9	10.4	4.0	10.7	1.2
A06—constipation	macrogol, lactulose	2.7	3.3	3.7	3.1	3.4	23.1	28.2	2.0
A07—antidiarrheals, intestinal anti‐inflammatory/anti‐infective agents	mesalazine, electrolytes	1.3	1.2	1.1	3.3	1.9	16.3	16.9	1.6
A10—diabetes	insulin aspart, metformin	0.1	0.6	2.0	5.8	2.7	43.7	84.7	4.4
B—blood									
B03—antianaemic preparations	ferrous sulphate, ferrous fumarate	3.4	3.9	1.8	5.4	3.7	16.4	26.9	1.5
C—cardiovascular system									
C01—cardiac Therapy	epinephrine, flecainide	2.7	9.9	13.2	9.6	10.8	21.7	33.8	1.4
C02—antihypertensives	clonidine, prazosin	0.1	1.4	6.4	5.0	4.2	64.5	72.6	3.9
C07—beta‐blocking agents	propranolol, atenolol	0.7	0.3	0.6	2.8	1.3	30.9	49.0	3.0
D—dermatologicals									
D01—antifungals for dermatological use	griseofulvin, terbinafine	1.2	1.3	1.3	1.7	1.4	7.6	30.0	1.5
D06—antibiotics and chemotherapeutics for dermatological use	silver sulfadiazine	1.1	1.6	1.2	1.7	1.5	1.1	4.4	1.1
D07—corticosteroids, dermatological preparations	hydrocortisone acetate, methylpredinosolone	131.4	88.4	53.3	51.0	68.8	7.4	29.3	1.6
D10—antiacne preparations	isoretinoin, adapalene + benzoyl peroxide	0.1	0.1	0.8	34.7	11.5	47.4	59.5	3.0
D11—other dermatological preparations	pimecrolimus	9.0	3.8	1.8	1.6	2.8	13.5	24.5	1.3
G—genitourinary system and sex hormones									
G03—sex hormones and modulators of the genital system	levonorgestrel + ethinylestradiol, etonogestrel	0.7	0.6	0.6	65.5	21.5	4.5	71.5	2.8
H—systemic hormones									
H01—pituitary and hypothalamic hormones and analogues	desmopressin, somatropin	0.1	0.2	4.9	2.8	2.7	29.5	72.5	4.5
H02—systemic corticosteroids	prednisolone sodium phosphate, prednisolone	134.2	164.1	73.3	33.4	92.7	3.6	32.4	1.6
H03—thyroid therapy	levothyroxine, carbimaxole	0.8	0.7	1.1	2.6	1.5	40.2	74.5	2.5
H04—pancreatic hormones	glucagon hydrochloride	0.1	0.3	1.1	2.1	1.2	49.5	49.1	1.7
J—systemic anti‐infectives									
J01—systemic antibacterials	amoxicillin, cephalexin	440.2	546.9	369.7	367.4	435.3	2.6	63.3	2.7
J05—systemic antivirals	acyclovir, famciclovir	0.1	0.2	0.6	2.5	1.1	7.3	15.7	1.5
M—Musculoskeletal system									
M01—anti‐inflammatory and antirheumatic products	ibuprofen, mefenamic acid	0.1	0.6	1.8	27.9	9.8	18.2	20.3	1.4
N—nervous system									
N02—analgesics	paracetamol + codeine, paracetamol	5.7	8.6	8.9	42.8	19.8	24.0	27.4	1.6
N03—antiepileptics	valproate, lamotrigine	1.2	2.3	4.2	6.7	4.4	43.4	83.7	8.8
N05—psycholeptics	risperidone, quetiapine	0.2	0.9	4.8	12.8	6.1	44.4	54.0	4.0
N06—psychoanaleptics	methylphenidate, fluoxetine	0.2	2.6	31.7	60.1	31.2	60.8	85.6	7.4
P—antiparasitics									
P03—ectoparasiticides	permethrin	2.4	3.1	3.0	3.8	3.4	1.6	37.9	1.5
R—respiratory system									
R03—obstructive airway diseases	salbutamol, fluticasone propionate	42.2	109.0	97.6	69.0	91.2	4.8	48.1	2.6
S—sensory organs									
S01—ophthalmologicals	chloramphenicol, fluorometholone	82.3	48.9	21.0	17.3	32.4	8.3	21.7	1.4
S02—otologicals	framycetin sulphate + gramicidin + dexamethasone	22.4	34.9	36.0	25.5	32.4	3.2	17.8	1.2
S03—ophthalmological and otological preparations	framycetin sulphate	9.6	5.4	2.2	1.4	3.4	2.5	9.5	1.1

Abbreviation: WHO ATC, World Health Organisation Anatomical Therapeutic Classification.

^a^ Among children with a first observed dispensing in 2013–2016 only,

^b^ the PBS classifies prochlorperazine as an antiemetic (not antipsychotic).

Among children <1 year, the top three classes with the highest average yearly prevalence were systemic antibacterials (440.2 children per 1000), primarily amoxicillin; systemic corticosteroids (134.2 per 1000), most commonly prednisolone; and topical corticosteroids (131.4 per 1000), most commonly hydrocortisone acetate (Table 2). The yearly prevalence of acid‐suppressing medicines was 47.2 per 1000 and much higher in this age group than any other. Children aged 1–5 years had the highest average yearly prevalence of systemic antibacterial use (546.9 children per 1000). Other common classes were systemic corticosteroids (164.1 per 1000), obstructive airway disease medicines (109.0 per 1000) and topical corticosteroids (88.4 per 1000) (Table 2). The prevalence of obstructive airway disease medicines peaked in children 1–5 years (Figure S1). Table 2 and Table S2 using age categories <2 years and 2–5 years are in Tables S3 and S4.

Similar patterns were observed for children aged 6–11 years, with the top three medicine classes including antibacterials (369.7 children per 1000), obstructive airway disease medicines (97.6 per 1000) and systemic corticosteroids (73.3 per 1000) (Table 2). The prevalence of psychostimulant use (27.0 per 1000), mostly methylphenidate, increased dramatically in this age group (Table S2). In older children (12–17 years), antibacterials were still the class with the highest average yearly prevalence (367.4 children per 1000), followed by obstructive airway disease medicines (69.0 per 1000); sex hormones (65.5 per 1000), primarily oral contraceptives; and psychoanaleptics (60.1 per 1000), mostly antidepressants and psychostimulants (Table 2). Dispensing of nervous system medicines (analgesics, antidepressants and antipsychotics) was highest in this age group (Table S2).

Dispensing rates (Figure S1) were generally similar in boys and girls across age groups, with a few exceptions. Sex hormones (i.e., hormonal contraceptives), iron preparations, antidepressants and anxiolytics were more common in older girls, while medicines to treat ADHD (clonidine and psychostimulants) and antipsychotics were more common in boys.

### 3.2 Patterns of use and prescriber type

Most (63.3%) of the children dispensed an antibacterial had more than one dispensing in the first year of observed use (mean 2.7 dispensings). Antiepileptics, antidepressants and psychostimulants had the greatest number of dispensings over one year (mean 8.8, 6.8 and 7.5, respectively) (Table S2). Overall, 87% (*n* = 5,232,501) of prescriptions was by a general practitioner (GP), 8.8% (*n* = 530,761) by a specialist, 4.3% (*n* = 258,883) by other (allied health, dentist) and 0.4% (*n* = 21,851) unknown. This varied by medicine class: systemic antiacne preparations (e.g., isotretinoin) and psychostimulants for ADHD were most likely to be prescribed by a specialist (95.2% and 83.9%) (Table S2). Antibiotics, antiparasitics and medicines to treat eye/ear infections were commonly prescribed by nonspecialists (Table 2).

### 3.3 Off‐label dispensing based on child's age at dispensing

Overall, the vast majority of dispensings (1759.4 dispensings per 1000 child‐years; 87.8%) were on‐label for the child's age (Table 3). Of off‐label dispensings (244.4 dispensings per 1000 child‐years; 12.2%), two‐thirds (66.3%) had age‐appropriate dosing recommendations in the AMH CDC. Off‐label use, with or without dosing recommendations, was highest in children aged <1 year (15.5% of dispensings) and 12–17 years (21.7%). Off‐label dispensing rates using the age categories <2 years and 2–5 years are in Table S5 and are similar to the primary analysis.

**TABLE 3:** On‐label and off‐label dispensing rates in children by age, 2013–17

	On‐label		Off‐label by age at dispensing		
	Dispensings per 1000 child‐years	Prescribed by specialist, %	Dispensings per 1000 child‐years	Prescribed by specialist, %	With age‐appropriate dose recommendations in prescribing guide, %
	*n* (% total)		*n* (% total)		
Age, years					
<1	1253.4 (84.5)	3.1	230.0 (15.5)	13.5	77.0
1–5	2058.1 (95.0)	3.2	107.5 (5.0)	18.6	64.6
6–11	1608.4 (91.8)	8.8	142.8 (8.2)	36.7	85.7
12–17	1738.2 (78.4)	13.1	478.5 (21.6)	18.9	59.6
All ages	1759.4 (87.8)	7.5	244.4 (12.2)	21.7	66.3

Note

On‐label = age‐appropriate dose recommendations in product information; Off‐label = no age‐appropriate dose recommendations in product information.

Among children <1 year, the highest rate of off‐label dispensing was for topical hydrocortisone acetate. Acid‐suppressing medicines, like omeprazole, for which use in children <1 year is off‐label, and ranitidine, whose use in children of any age is off‐label, were also common, making up 35.5% of off‐label dispensings (Table 4; Table S6). Nearly all (98.5%) acid‐suppressing medicine dispensings were off‐label for children <1 year (Table S7). Among off‐label dispensings, the most commonly dispensed medicines without age‐appropriate dose recommendations in the AMH CDC were ranitidine, pantoprazole and lansoprazole in children <6 months; and topical methylprednisolone, which is not recommended in children <4 months (Table 4). Oral liquid salbutamol was also commonly dispensed even though it is not recommended at any age. In children aged 6–11 years, commonly off‐label medicines were clonidine, antidepressants (e.g., fluoxetine) and antipsychotics (e.g., risperidone). Clonidine, risperidone and fluoxetine represented 16.0%, 13.2% and 12.6% of all off‐label dispensings in this age group (Table 4). However, these medicines all had age‐appropriate dose recommendations in the AMH CDC. In children 12–17 years, the most common off‐label medicines were oral contraceptives (i.e., levonorgestrel + ethinylestradiol).

**TABLE 4:** Most common medicines dispensed off‐label by presence of age‐appropriate recommended doses in prescribing guide, Australian Medicines Handbook Children's Dosing Companion (AMH CDC). Some medicines may fall into both categories for a given age group depending on the cut‐offs for dose recommendations as listed in Table S6

Age group	Off‐label with age‐appropriate recommended doses in prescribing guide (AMH CDC)			Off‐label without age‐appropriate recommended doses in prescribing guide (AMH CDC)		
	Medicine name and route of administration	Dispensings per 1000 child‐years	Prescribed by specialist, %	Medicine name and route of administration	Dispensings per 1000 child‐years	Prescribed by specialist, %
<1 year	TOTAL	177.2	12.1	TOTAL	52.8	18.9
	hydrocortisone acetate (topical)	76.1	5.7	ranitidine (oral)	17.4	21.2
	omeprazole (oral)	52.7	23.9	methylprednisolone (topical)	6.0	13.9
	framycetin sulphate (eye drops)	10.4	0.6	salbutamol (oral liquid)	5.8	0.3
	salbutamol (inhaled)	5.4	1.7	fluticasone propionate (inhaled)	4.9	25.4
	azithromycin (oral)	5.3	6.2	lansoprazole (oral)	4.5	30.3
	ranitidine (oral)	5.3	21.4	roxithromycin (oral)	3.9	2.8
	timolol (eye drops)	4.7	67.8	pantoprazole (oral)	1.7	47.2
	ondansetron (oral)	3.5	2.1	topiramate (oral)	1.1	32.7
	epinephrine (auto‐injector)	2.6	58.3	dexamethasone (eye drops)	0.7	43.4
	ipratropium (inhaled)	1.6	5.5	ipratropium (inhaled)	0.5	4.0
1–5 years	TOTAL	69.4	16.8	TOTAL	38.1	22.7
	hydrocortisone acetate (topical)	29.4	3.6	fluticasone +salmeterol (inhaled)	13.3	8.6
	framycetin sulphate (eye drops)	6.0	2.3	salbutamol (oral liquid)	6.8	0.4
	clonidine (oral)	3.1	70.6	methylphenidate (oral)	3.6	88.6
	ondansetron (oral)	2.6	1.6	dexamfetamine (oral)	1.1	90.2
	levetiracetam (oral)	2.5	44.2	pantoprazole (oral)	1.1	57.8
	oxycodone (oral)	2.3	67.7	fluoxetine (oral)	0.8	15.1
	ipratropium (inhaled)	2.1	4.0	risperidone (oral)	0.8	81.3
	ranitidine (oral)	1.8	12.1	beclomethasone (inhaled)	0.8	77.5
	hydrocortisone (oral)	1.6	44.9	budesonide +formoterol (inhaled)	0.7	9.4
	epinephrine (auto‐injector)	1.5	53.2	ciclesonide (inhaled)	0.5	42.0
6–11 years	TOTAL	122.4	28.2	TOTAL	20.4	28.4
	clonidine (oral)	22.9	69.1	salbutamol (oral liquid)	1.8	0.4
	risperidone (oral)	18.9	70.0	escitalopram (oral)	1.2	48.5
	fluoxetine (oral)	18.0	70.2	oxybutynin (patch)	1.0	11.3
	hydrocortisone acetate (topical)	11.4	4.3	fluoxetine (oral)	1.0	82.9
	levetiracetam (oral)	4.9	42.5	paracetamol/codeine (oral)	1.0	61.8
	budesonide +formoterol (inhaled)	4.4	9.3	calcipotriol +betamethasone (topical)	0.9	36.9
	amitriptyline (oral)	3.9	45.2	imipramine (oral)	0.8	30.8
	fluorometholone (eye drops)	2.8	25.1	ramipril (oral)	0.6	41.1
	oxycodone (oral)	2.5	49.0	perindopril (oral)	0.5	27.0
	ranitidine (oral)	2.5	8.3	citalopram (oral)	0.5	58.1
12–17 years	TOTAL	285.1	26.3	TOTAL	193.4	10.8
	fluoxetine (oral)	90.4	33.4	levonorgestrel +ethinylestradiol (oral)	101.9	2.5
	minocycline (oral)	45.2	11.5	desvenlafaxine (oral)	8.7	23.1
	escitalopram (oral)	25.1	18.9	medroxyprogesterone (injection)	6.9	1.9
	clonidine (oral)	18.5	61.3	venlafaxine (oral)	6.6	24.5
	risperidone (oral)	12.8	58.8	norethisterone +ethinylestradiol (oral)	6.5	6.1
	amitriptyline (oral)	9.3	28.4	mirtazapine (oral)	6.5	26.6
	hydrocortisone acetate (topical)	8.0	6.5	norethisterone (oral)	4.1	8.8
	citalopram (oral)	6.9	19.5	duloxetine (oral)	3.7	24.0
	diclofenac (oral)	5.2	13.2	meloxicam (oral)	3.5	23.0
	olanzapine (oral)	4.0	36.3	rabeprazole (oral)	3.0	11.1

In general, dispensings considered off‐label by age were more likely to be prescribed by a specialist (21.7%) than on‐label dispensings (7.5%) (Table 3). Psychotropic medicines as well as clonidine had high rates of off‐label prescribing by a specialist, especially in younger children.

## 4 COMMENT

### 4.1 Principal findings

In this whole‐of‐population study of Australian children, we observed an average of two medicine dispensings per child per year, dominated by antibacterials. Aside from antibacterials, in younger children, common medicine classes were corticosteroids (systemic and topical) and acid‐suppressing medicines, with a shift towards greater use of respiratory medicines and medicines to treat ADHD in school age children, and oral contraceptives and psychotropics in adolescents. While 12% of dispensings was considered off‐label by age, two‐thirds of these had contemporary advice on age‐appropriate paediatric dosing in a national prescribing resource. While off‐label dispensing of medicines without specific dose recommendations in children was a small proportion of overall use, it was concentrated in a few classes, such as those to treat gastro‐oesophageal reflux disease and psychotropic medicines.

### 4.2 Strengths of the study

While many recent previous studies focussed on small or select samples (e.g., inpatients), we have dispensing data on a representative sample of 1 in 7 children in a mostly outpatient setting over 6 years, allowing us to make robust inference about medicine use in the whole population. We have quantified for the first time the extent of prescribed medicine use in Australian children by age and identified areas of concern, including high rates of prescribing of antibacterials, acid‐suppressing medicines in young children and psychotropic medicines in older children, which warrant further investigation to understand drivers of these patterns of use. Given that not all off‐label is necessarily inappropriate, we also took our analysis one step further by referring to an independent prescribing resource to determine which off‐label dispensing was supported by evidence.

### 4.3 Limitations of the data

These findings do not apply to other settings, such as in hospitals, where off‐label prescribing is likely to be higher.^30^, ^31^ Off‐label prescribing has also been shown to be high in newborns, especially in intensive care units^32^, ^33^; however, we were unable to explore this population due to the lack of exact date of birth and the incomplete capture of medicine use in hospitals. More in‐depth studies of this population in the Australian context are warranted. We did not have information on indication for prescribing, and thus, our estimates likely represent the lower bound for off‐label prescribing. Furthermore, for medicines where recommendations were based on body weight, we relied on population‐level weight estimates; however, this applied to only 1.8% of formulations in our data. Medicines dispensed are not necessarily taken, and we do not have data on medicines not dispensed through the PBS. The volume of private prescribing in children is unknown but varies by medicine; for instance, general practice data showed that <1% of amoxicillin is privately prescribed.^34^

### 4.4 Interpretation

In our study, on‐label prescribing was driven by antibiotics, representing nearly half of all dispensings. We found that 435 per 1000 children were dispensed a systemic antibiotic per year with nearly two‐thirds having multiple dispensings in a year. This is comparable to contemporary rates from France (405 per 1000 children),^17^ Germany (428 per 1000 children),^35^ New Zealand (480 per 1000 children)^36^ and Finland (375 per 1000 children).^37^ Antibiotic overuse is a pervasive problem^38^, ^39^ and their prescribing for common childhood conditions (e.g., otitis media) is considered low‐value care owing to a lack of evidence and risk of side effects.^40^, ^41^ While we were unable to assess indication for prescribing, a study of Australian general practice (2015–17) found that nearly all diagnosed cases of otitis media and tonsillitis and two‐thirds of acute upper respiratory tract infections were treated with antibiotics, despite guidelines recommending their use in a minority of cases.^34^, ^38^ This contrasts with the Netherlands, with 55% and 14% of otitis media and upper respiratory tract infection episodes resulting in an antibiotic prescription,^42^ and Sweden with 25% of upper respiratory tract infection episodes treated with an antibiotic.^43^

Uncomplicated gastro‐oesophageal reflux in infants, a normal physiological condition typically not requiring treatment, has also been highlighted as a commonly over‐treated condition.^40^ PPIs and other acid‐suppressing medicines are often prescribed to treat refluxing infants, in addition to nonspecific symptoms such as irritability, but there is no robust evidence of efficacy in very young children.^44^ This contrasts with gastro‐oesophageal reflux disease (GORD), which is more serious, and may require pharmacotherapy.^45^ We observed dispensing of acid‐suppressing medicines to 4.7% of children <1 year, similar to rates from New Zealand (5.7%) and Ireland (4.5%).^13^, ^46^ While symptoms of reflux‐like regurgitation affect roughly half of children <3 months,^47^ a 2018 study of 1000 Australian general practices found that 2.7% of infants <1 year had a diagnosis of reflux or GORD, with roughly half prescribed an acid‐suppressing medicine.^48^

The PI may not always reflect the most current evidence, which may explain much off‐label prescribing. For instance, topical hydrocortisone was one of the most common off‐label medicines dispensed. Atopic dermatitis is relatively common especially in children <2 years, with a 2020 study of Australian patients in general practice reporting an estimated lifetime prevalence in children ≤4 years at 19%.^49^ Therefore, while the PI includes no specific recommendations in children, it is a mild corticosteroid and its use is recommended in children as long as care is taken due to the risk of adverse effects associated with increased skin absorption.^50^

Other common medicines prescribed off‐label included psychotropic medicines, especially ADHD medicines, antidepressants and antipsychotics in young children below the minimum recommended age.^12^ Misdiagnosis and overtreatment of children who are youngest in their grade with ADHD has been observed in many jurisdictions including Australia.^51^ Furthermore, many antidepressants offer little benefit in children with major depression but are associated with adverse effects including suicidal ideation.^52^ However, the most commonly dispensed antidepressant in our study was fluoxetine, which has the most evidence supporting its use in this population.^52^ Concerningly, increasing psychotropic‐related self‐harm has been observed in Australian children.^16^

Estimates of off‐label prescribing in outpatient settings vary greatly, depending on the definition; a 2018 systematic review found rates ranging from 1% to 62%.^5^ We have defined off‐label use based on age and route of administration and thus have likely underestimated off‐label use, with other studies finding high rates of off‐label prescribing in terms of daily dose and indication.^7^, ^30^ A 2019 US study^6^ found that 18% of off‐label medicines was off‐label by age, compared with 85% off‐label by indication. While most off‐label use in our study involved prescribing below the recommended minimum age, we also identified use of medicines contraindicated in children. International and local guidelines advise against use of oral salbutamol, due to slower onset of action and greater side effects.^53^ Yet, it was one of the most common medicines prescribed in our data without age‐appropriate dose recommendations. However, for some medicines, the absence of age‐appropriate dose recommendations does not necessarily imply that they should not be used. Older teenagers may be physiologically similar to adults. While oral contraceptives did not have any specific dose recommendations for girls <18 years at the time of this study, adult doses are generally considered appropriate in girls postmenarche.

## 5 CONCLUSIONS

We demonstrated that prescribed medicine use is common in children, dominated by concerningly high rates of antibacterial prescribing, with 1 in 24 dispensings having no contemporary advice on age‐appropriate paediatric dosing. While not all off‐label dispensing by age is problematic, it is worth nothing that these were concentrated in medicine classes such as acid‐suppressing medicines and psychotropics where there are currently concerns about overprescribing and increasing harms.^16^, ^54^ Our findings highlight a need for more evidence in this population to support best‐practice prescribing, minimise low‐value care and improve outcomes.

## CONFLICT OF INTEREST

AS, CB, ML, HZ and SAP are employees of the Centre for Big Data Research in Health, UNSW Sydney which received funding in 2020 from AbbVie Australia to conduct research, unrelated to the submitted work. AbbVie did not have any knowledge of, or involvement in, the present study. SAP is a member of the Drug Utilisation Sub Committee of the Pharmaceutical Benefits Advisory Committee. The views expressed in this paper do not represent those of the Committee.

## AUTHOR CONTRIBUTIONS

All authors contributed to the conception and design of the work, and the interpretation of data. SAP and NN acquired the data. AS, ML and CB analysed the data. AS drafted the work, and all authors revised it critically. All authors approved the version to be published and agree to be accountable for all aspects of the work.

## Supporting information

Supplementary Material

## Data Availability

The data in this study were used under license from the Australian Government Services, Australia, and restrictions apply to their availability. Access to these data by other individuals or authorities is not permitted without the express permission of the approving human research ethics committees and data custodians. Interested parties can contact the Australian Government Services, Australia (https://www.servicesaustralia.gov.au/medicare‐statistics).
