# Supplementary material for: Prescribed medicine use and extent of off‐label use according to age in a nationwide sample of Australian children
Source: Paediatr Perinat Epidemiol. 2022 Feb 16;36(5):726–37. doi: 10.1111/ppe.12870 (PMC9540111; doi:10.1111/ppe.12870)

Supplemental Tables

eTable 1. Characteristics of study population using alternate age categories, 2013-2017

	No. children with ≥1 dispensings	Child-years	Dispensings	Dispensings per child-year
**Total**	840,190	4,026,231	8,219,722	2.0
**Age**				
<2 years	209,125	466,529	995,716	2.1
2-5 years	303,084	932,305	1,951,555	2.1
6-11 years	332,607	1,340,014	2,398,899	1.8
12-17 years	323,085	1,281,384	2,904,068	2.3

eTable 2. Prevalence by World Health Organisation Anatomic Therapeutical Chemical (ATC) chemical subgroup, restricted to subgroups with an average yearly prevalence ≥1 per 1000 children, 2013-17

ATC Classification	Most common examples of medicines in subgroup	Average yearly prevalence per 1000 children					Prescribed by specialist, %	Dispensings per child in first year of observed use[a]	
		<1	1-5	6-11	12-17	All (0-17)		Two or more, %	Mean
**A – Alimentary Tract**									
A01A – Stomatological preparations	nystatin, amphotericin b	13.0	2.8	0.9	1.2	2.3	2.2	15.2	1.2
A02B – Drugs for peptic ulcer and gastro-oesophageal reflux disease	omeprazole, esomeprazole	47.0	4.9	7.8	19.3	13.0	18.1	35.8	2.2
A03F – Propulsives	metoclopramide, domperidone	0.3	0.4	1.5	12.5	4.7	4.1	11.6	1.2
A04A – Antiemetics and antinauseants	ondansetron, prochlorperazine[b]	3.6	12.7	9.5	9.9	10.4	4.0	10.7	1.2
A06A – Drugs for constipation	macrogol, lactulose	2.7	3.3	3.7	3.1	3.4	23.1	28.2	2.0
A10A – Insulins and analogues	insulin aspart, insulin glargine	0.0	0.5	1.8	4.0	2.1	46.0	96.3	4.7
**B – Blood**									
B03A – Iron preparations	ferrous sulfate, ferrous fumarate	3.2	3.8	1.6	4.0	3.1	16.9	26.1	1.5
**C – Cardiovascular**									
C01C – Cardiac stimulants excluding cardiac glycosides	epinephrine	2.6	9.8	13.1	9.4	10.7	21.6	33.7	1.4
C02A – Antiadrenergic agents, centrally acting	clonidine	0.0	1.3	6.4	4.9	4.2	66.3	72.5	3.7
C07A – Beta blocking agents	propranolol, atenolol	0.7	0.3	0.6	2.8	1.3	30.9	49.0	3.0
**D – Dermatologicals**									
D06B – Chemotherapeutics for topical use	silver sulfadiazine	1.1	1.6	1.2	1.7	1.5	1.1	4.4	1.1
D07A – Corticosteroids, plain	methylprednisolone, mometasone	131.4	88.4	53.3	51.0	68.8	7.4	29.3	1.6
D10A – Anti-acne preparations for topical use	adapalene + benzoyl peroxide	0.0	0.0	0.8	24.3	8.1	12.6	44.3	2.0
D10B – Anti-acne preparations for systemic use	isotretinoin	0.0	0.0	0.1	12.3	4.0	95.2	92.0	5.2
D11A – Other dermatological preparations	pimecrolimus	9.0	3.8	1.8	1.6	2.8	13.5	24.5	1.3
**G – Genitourinary And Sex Hormones**									
G03A – Hormonal contraceptives for systemic use	levonorgestrel + ethyinylestradiol, etonogestrel	0.2	0.2	0.4	63.1	20.5	2.7	72.4	2.8
**H – Systemic Hormones**									
H01B – Posterior pituitary lobe hormones	desmopressin	0.0	0.1	4.6	2.6	2.5	23.8	70.9	4.5

ATC Classification	Most common examples of medicines in subgroup	Average yearly prevalence per 1000 children					Prescribed by specialist, %	Dispensings per child in first year of observed use[a]	
		<1	1-5	6-11	12-17	All (0-17)		Two or more, %	Mean
H02A – Corticosteroids for systemic use, plain	prednisolone, prednisone	134.2	164.1	73.3	33.4	92.7	3.6	32.4	1.6
H03A – Thyroid preparations	levothyroxine	0.8	0.7	1.0	2.3	1.4	39.4	73.8	2.3
H04A – Glycogenolytic hormones	glucagon hydrochloride	0.1	0.3	1.1	2.1	1.2	49.5	49.1	1.7
**J – Systemic Antiinfectives**									
J01A – Tetracyclines	doxycycline, minocycline	0.1	0.2	1.4	53.3	17.7	8.2	65.7	3.9
J01C – Beta-lactam antibacterials, penicillins	amoxicillin, amoxicillin + clavulanic acid	358.3	428.5	256.3	217.1	307.4	2.1	53.1	2.1
J01D – Other beta-lactam antibacterials	cefalexin, cefaclor	100.4	180.7	123.2	110.9	138.2	2.6	38.9	1.7
J01E – Sulfonamides and trimethoprim	trimethoprim + sulfamethoxazole, trimethoprim	18.4	23.9	13.4	15.6	17.8	7.0	29.8	1.6
J01F – Macrolides, lindosamines and streptogramins	erythromycin,, roxithromycin	47.8	81.7	55.6	66.7	68.1	2.5	34.3	1.6
J01X – Other antibacterials	metronidazole, tinidazole	2.7	8.0	7.2	10.0	8.3	6.4	15.7	1.2
J05A – Direct acting antivirals	famciclovir, valaciclovir	0.1	0.2	0.6	2.5	1.1	7.3	15.0	1.2
**M – Musculoskeletal**									
M01A – Antiinflammatory and antirheumatic products, non-steroids	ibuprofen, mefenamic acid	0.1	0.6	1.7	27.6	9.7	17.6	20.1	1.4
**N – Nervous System**									
N02A – Opioids	paracetamol + codeine, oxycodone	0.5	2.9	3.5	35.7	13.6	30.0	22.1	1.4
N02B – Other analgesics and antipyretics	paracetamol, pregabalin[b]	5.1	5.6	4.4	4.3	4.9	7.1	25.5	1.6
N02C – Antimigraine preparations	sumatriptan, pizotifen	0.0	0.3	1.2	5.2	2.2	20.1	41.5	2.2
N03A – Antiepileptics	valproate, lamotrigine	1.2	2.3	4.2	6.7	4.4	43.4	83.7	8.8
N05A – Antipsychotics	risperidone, quetiapine	0.0	0.7	4.2	7.8	4.2	54.8	79.2	5.8
N05B – Anxiolytics	diazepam, oxazepam	0.1	0.2	0.6	4.0	1.6	15.3	21.9	1.6
N06A – Antidepressants	fluoxetine, sertraline	0.2	0.8	6.9	40.3	15.6	33.2	80.6	6.8
N06B – Psychostimulants, agents used for ADHD and nootropics	methylphenidate, lisdexamfetamine	0.0	1.9	27.0	24.0	17.7	83.9	91.1	7.5
**P – Antiparasitics**									
P03A – Ectoparasiticides, including scabicides	permethrin	2.4	3.1	3.0	3.8	3.4	1.6	37.9	1.5
**R – Respiratory**									

ATC Classification	Most common examples of medicines in subgroup	Average yearly prevalence per 1000 children <1	1-5	6-11	12-17	All (0-17)	Prescribed by specialist, %	Dispensings per child in first year of observed use[a] Two or more, %	Mean
R03A – Adrenergics, inhalants	salbutamol, fluticasone + salmeterol	33.6	78.4	71.0	59.4	69.2	3.1	36.1	1.9
R03B – Other drugs for obstructive airway diseases, inhalants	fluticasone, ipratropium	6.9	43.4	40.7	15.8	32.6	7.1	48.5	2.2
R03C – Adrenergics for systemic use	salbutamol	5.6	5.7	1.4	0.3	2.6	0.5	14.8	1.2
R03D – Other systemic drugs for obstructive airway disease	montelukast, theophylline	0.2	13.8	14.1	4.0	10.3	10.5	65.8	4.4
**S – Sensory Organs**									
S01A – Ophthalmological antiinfectives	chloramphenicol, tobramycin	78.1	44.8	16.0	12.3	27.6	2.3	18.7	1.2
S01B – Ophthalmological antiinflammatory agents	hydrocortisone acetate, fluorometholone	2.3	3.2	4.2	4.5	4.0	29.2	19.2	1.5
S02A – Otological antiinfectives	ciprofloxacin, chloramphenicol	1.4	3.0	1.5	0.6	1.7	10.5	26.0	1.4
S02C – Otological corticosteroids and antiinfectives in combination	framycetin sulfate + gramicidin + dexamethasone, triamcinolone + neomycin + gramicidin + nystatin	21.2	32.2	34.8	25.1	31.0	2.8	16.8	1.2
S03A – Ophthalmological and otological antiinfectives	framycetin sulfate	9.6	5.4	2.2	1.4	3.4	2.5	9.5	1.1

[a]Restricted to children with a first dispensing in 2013-2016

[b]The Australian Pharmaceutical Benefits Scheme classifies prochlorperazine as an antiemetic (not antipsychotic) and pregabalin as an analgesic (not antiepileptic)

eTable 3. Prevalence by WHO ATC therapeutic subgroup using alternate age categories, restricted to subgroups with an average yearly prevalence ≥1 per 1000 children, 2013-17

Therapeutic subgroup	Most common examples of medicines in subgroup	Average yearly prevalence per 1000 children				
		**<2**	**2-5**	**6-11**	**12-17**	**All (0-17)**
**A – Alimentary Tract**						
A01 – Stomatological Preparations	nystatin, amphotericin B	9.3	2.0	0.9	1.2	2.3
A02 – Acid Related Disorders	omeprazole, esomeprazole	26.0	4.4	8.0	19.3	13.1
A03 – Functional Gastrointestinal Disorders	metoclopramide, domperidone	0.3	0.5	1.6	12.5	4.7
A04 – Antiemetics And Antinauseants	ondansetron, prochlorperazine[b]	7.7	13.1	9.5	9.9	10.4
A06 – Constipation	macrogol, lactulose	2.8	3.5	3.7	3.1	3.4
A07 – Antidiarrheals, Intestinal Antiinflammatory/Antiinfective Agents	mesalazine, electrolytes	1.6	1.1	1.1	3.3	1.9
A10 – Diabetes	insulin aspart, metformin	0.1	0.7	2.0	5.8	2.7
**B – Blood**						
B03 – Antianaemic Preparations	ferrous sulfate, ferrous fumarate	4.1	3.7	1.8	5.4	3.7
**C – Cardiovascular system**						
C01 – Cardiac Therapy	epinephrine, flecainide	4.7	10.7	13.2	9.6	10.8
C02 – Antihypertensives	clonidine, prazosin	0.1	1.7	6.4	5.0	4.2
C07 – Beta Blocking Agents	propranolol, atenolol	0.5	0.2	0.6	2.8	1.3
**D – Dermatologicals**						
D01 – Antifungals For Dermatological Use	griseofulvin, terbinafine	1.1	1.3	1.3	1.7	1.4
D06 – Antibiotics And Chemotherapeutics For Dermatological Use	silver sulfadiazine	2.0	1.3	1.2	1.7	1.5
D07 – Corticosteroids, Dermatological Preparations	hydrocortisone acetate, methylpredinosolone	124.7	80.3	53.3	51.0	68.8
D10 – Anti-Acne Preparations	isoretinoin, adapalene + benzoyl peroxide	0.0	0.1	0.8	34.7	11.5
D11 – Other Dermatological Preparations	pimecrolimus	7.4	3.2	1.8	1.6	2.8
**G – Genitourinary system & sex hormones**						
G03 – Sex Hormones And Modulators Of The Genital System	levonorgestrel + ethinylestradiol, etonogestrel	0.7	0.6	0.6	65.5	21.5
**H – Systemic hormones**						
H01 – Pituitary And Hypothalamic Hormones And Analogues	desmopressin, somatropin	0.1	0.2	4.9	2.8	2.7
H02 – Systemic corticosteroids	prednisolone sodium phosphate, prednisolone	169.5	155.0	73.3	33.4	92.7
H03 – Thyroid Therapy	levothyroxine, carbimaxole	0.7	0.8	1.1	2.6	1.5
H04 – Pancreatic Hormones	glucagon hydrochloride	0.1	0.4	1.1	2.1	1.2
**J – Systemic antiinfectives**						
J01 – Systemic antibacterials	amoxicillin, cephalexin	520.2	533.8	369.7	367.4	435.3
J05 – Systemic antivirals	acyclovir, famciclovir	0.1	0.2	0.6	2.5	1.1
**M – Musculoskeletal system**						

Therapeutic subgroup	Most common examples of medicines in subgroup	Average yearly prevalence per 1000 children				
		<2	2-5	6-11	12-17	All (0-17)
M01 – Antiinflammatory and Antirheumatic Products	ibuprofen, mefenamic acid	0.2	0.7	1.8	27.9	9.8
**N – Nervous system**						
N02 – Analgesics	paracetamol + codeine, paracetamol	6.3	9.0	8.9	42.8	19.8
N03 – Antiepileptics	valproate, lamotrigine	1.4	2.6	4.2	6.7	4.4
N05 – Psycholeptics	risperidone, quetiapine	0.2	1.1	4.8	12.8	6.1
N06 – Psychoanaleptics	methylphenidate, fluoxetine	0.2	3.2	31.7	60.1	31.2
**P – Antiparasitics**						
P03 – Ectoparasiticides	permethrin	2.9	3.1	3.0	3.8	3.4
**R – Respiratory system**						
R03 – Obstructive Airway Diseases	salbutamol, fluticasone propionate	69.3	114.3	97.6	69.0	91.2
**S – Sensory organs**						
S01 – Ophthalmologicals	chloramphenicol, fluorometholone	83.1	39.6	21.0	17.3	32.4
S02 - Otologicals	framycetin sulfate + gramicidin + dexamethasone	27.0	35.7	36.0	25.5	32.4
S03 – Ophthalmological and otological preparations	framycetin sulfate	9.6	4.2	2.2	1.4	3.4

[a]Among children with a first observed dispensing in 2013-2016 only

[b]The PBS classifies prochlorperazine as an antiemetic (not antipsychotic)

WHO ATC = World Health Organisation Anatomic Therapeutic Chemical

eTable 4. Prevalence by World Health Organisation Anatomic Therapeutical Chemical (ATC) chemical subgroup using alternative age categories, restricted to subgroups with an average yearly prevalence ≥1 per 1000 children, 2013-17

ATC Classification	Most common examples of medicines in subgroup	Average yearly prevalence per 1000 children				
		<2	2-5	6-11	12-17	All (0-17)
**A – Alimentary Tract**						
A01A – Stomatological preparations	nystatin, amphotericin b	9.3	2.0	0.9	1.2	2.3
A02B – Drugs for peptic ulcer and gastro-oesophageal reflux disease	omeprazole, esomeprazole	25.9	4.3	7.8	19.3	13.0
A03F – Propulsives	metoclopramide, domperidone	0.3	0.5	1.5	12.5	4.7
A04A – Antiemetics and antinauseants	ondansetron, prochlorperazine[b]	7.7	13.1	9.5	9.9	10.4
A06A – Drugs for constipation	macrogol, lactulose	2.8	3.5	3.7	3.1	3.4
A10A – Insulins and analogues	insulin aspart, insulin glargine	0.1	0.6	1.8	4.0	2.1
**B – Blood**						
B03A – Iron preparations	ferrous sulfate, ferrous fumarate	3.9	3.6	1.6	4.0	3.1
**C – Cardiovascular**						
C01C – Cardiac stimulants excluding cardiac glycosides	epinephrine	4.7	10.7	13.1	9.4	10.7
C02A – Antiadrenergic agents, centrally acting	clonidine	0.1	1.7	6.4	4.9	4.2
C07A – Beta blocking agents	propranolol, atenolol	0.5	0.2	0.6	2.8	1.3
**D – Dermatologicals**						
D06B – Chemotherapeutics for topical use	silver sulfadiazine	2.0	1.3	1.2	1.7	1.5
D07A – Corticosteroids, plain	methylprednisolone, mometasone	124.7	80.3	53.3	51.0	68.8
D10A – Anti-acne preparations for topical use	adapalene + benzoyl peroxide	0.0	0.0	0.8	24.3	8.1
D10B – Anti-acne preparations for systemic use	isotretinoin	0.0	0.0	0.1	12.3	4.0
D11A – Other dermatological preparations	pimecrolimus	7.4	3.2	1.8	1.6	2.8
**G – Genitourinary And Sex Hormones**						
G03A – Hormonal contraceptives for systemic use	levonorgestrel + ethyinylestradiol, etonogestrel	0.2	0.2	0.4	63.1	20.5
**H – Systemic Hormones**						
H01B – Posterior pituitary lobe hormones	desmopressin	0.0	0.1	4.6	2.6	2.5
H02A – Corticosteroids for systemic use, plain	prednisolone, prednisone	169.5	155.0	73.3	33.4	92.7
H03A – Thyroid preparations	levothyroxine	0.7	0.7	1.0	2.3	1.4
H04A – Glycogenolytic hormones	glucagon hydrochloride	0.1	0.4	1.1	2.1	1.2
**J – Systemic Antiinfectives**						
J01A – Tetracyclines	doxycycline, minocycline	0.1	0.2	1.4	53.3	17.7
J01C – Beta-lactam antibacterials, penicillins	amoxicillin, amoxicillin + clavulanic acid	431.5	407.3	256.3	217.1	307.4

ATC Classification	Most common examples of medicines in subgroup	Average yearly prevalence per 1000 children				
		<2	2-5	6-11	12-17	All (0-17)
J01D – Other beta-lactam antibacterials	cefalexin, cefaclor	148.9	178.1	123.2	110.9	138.2
J01E – Sulfonamides and trimethoprim	trimethoprim + sulfamethoxazole, trimethoprim	23.3	22.9	13.4	15.6	17.8
J01F – Macrolides, lindosamines and streptogramins	erythromycin,, roxithromycin	70.9	79.2	55.6	66.7	68.1
J01X – Other antibacterials	metronidazole, tinidazole	5.5	8.0	7.2	10.0	8.3
J05A – Direct acting antivirals	famciclovir, valaciclovir	0.1	0.2	0.6	2.5	1.1
**M – Musculoskeletal**						
M01A – Antiinflammatory and antirheumatic products, non-steroids	ibuprofen, mefenamic acid	0.2	0.7	1.7	27.6	9.7
**N – Nervous System**						
N02A – Opioids	paracetamol + codeine, oxycodone	0.8	3.3	3.5	35.7	13.6
N02B – Other analgesics and antipyretics	paracetamol, pregabalin[b]	5.4	5.5	4.4	4.3	4.9
N02C – Antimigraine preparations	sumatriptan, pizotifen	0.1	0.3	1.2	5.2	2.2
N03A – Antiepileptics	valproate, lamotrigine	1.4	2.6	4.2	6.7	4.4
N05A – Antipsychotics	risperidone, quetiapine	0.0	0.8	4.2	7.8	4.2
N05B – Anxiolytics	diazepam, oxazepam	0.1	0.2	0.6	4.0	1.6
N06A – Antidepressants	fluoxetine, sertraline	0.2	1.0	6.9	40.3	15.6
N06B – Psychostimulants, agents used for ADHD and nootropics	methylphenidate, lisdexamfetamine	0.0	2.3	27.0	24.0	17.7
**P – Antiparasitics**						
P03A – Ectoparasiticides, including scabicides	permethrin	2.9	3.1	3.0	3.8	3.4
**R – Respiratory**						
R03A – Adrenergics, inhalants	salbutamol, fluticasone + salmeterol	54.4	80.1	71.0	59.4	69.2
R03B – Other drugs for obstructive airway diseases, inhalants	fluticasone, ipratropium	17.8	48.3	40.7	15.8	32.6
R03C – Adrenergics for systemic use	salbutamol	7.4	4.9	1.4	0.3	2.6
R03D – Other systemic drugs for obstructive airway disease	montelukast, theophylline	1.1	16.9	14.1	4.0	10.3
**S – Sensory Organs**						
S01A – Ophthalmological antiinfectives	chloramphenicol, tobramycin	79.5	35.3	16.0	12.3	27.6
S01B – Ophthalmological antiinflammatory agents	hydrocortisone acetate, fluorometholone	2.5	3.3	4.2	4.5	4.0
S02A – Otological antiinfectives	ciprofloxacin, chloramphenicol	2.5	2.9	1.5	0.6	1.7
S02C – Otological corticosteroids and antiinfectives in combination	framycetin sulfate + gramicidin + dexamethasone, triamcinolone + neomycin + gramicidin + nystatin	24.9	33.2	34.8	25.1	31.0

ATC Classification	Most common examples of medicines in subgroup	Average yearly prevalence per 1000 children				
		<2	2-5	6-11	12-17	All (0-17)
S03A – Ophthalmological and otological antiinfectives	framycetin sulfate	9.6	4.2	2.2	1.4	3.4

[a]Restricted to children with a first dispensing in 2013-2016

[b]The Australian Pharmaceutical Benefits Scheme classifies prochlorperazine as an antiemetic (not antipsychotic) and pregabalin as an analgesic (not antiepileptic)

eTable 5. On-label and off-label dispensing rate in children by age using alternate age categories, 2013-17

	**On-label**		**Off-label by age at dispensing**		
	**Dispensings per 1000 child-years**		**Dispensings per 1000 child-years**		
	**n (% total)**	**Prescribed by specialist, %**	**n (% total)**	**Prescribed by specialist, %**	**With age-appropriate dose recommendations in prescribing guide, %**
**Age group**					
<2 years	1854.2 (91.1)	2.7	181.3 (8.9)	12.6	78.0
2-5 years	1959.4 (95.1)	3.4	101.1 (4.9)	21.6	59.6
6-11 years	1608.4 (91.8)	8.8	142.8 (8.2)	36.7	85.7
12-17 years	1738.2 (78.4)	13.1	478.5 (21.6)	18.9	59.6
All ages	1759.4 (87.8)	7.5	244.4 (12.2)	21.7	66.3

On-label = age-appropriate dose recommendations in product information; Off-label = no age-appropriate dose recommendations in Product Information

eTable 6. Indications and dose recommendation for commonly dispensed off-label medicines listed in Table 4

WHO ATC code	Medicine name and route of administration	Primary indications	Age and/or weight range for dose recommendations based on AMH CDC		
			**None**	**Off-label**	**On-label**
A02BA02	ranitidine (oral)	gastro-oesophageal reflux disease	<6 mos	≥6 mos	--
A02BC01	omeprazole (oral)	gastro-oesophageal reflux disease	<1 yr	--	≥1 yr
A02BC02	pantoprazole (oral)	gastro-oesophageal reflux disease	<5 yrs	--	≥5 yrs
A02BC03	lansoprazole (oral)	gastro-oesophageal reflux disease	<1 yr	≥1 yr	--
A02BC04	rabeprazole (oral)	gastro-oesophageal reflux disease	All ages	--	--
A04AA01	ondansetron (oral)	nausea/vomiting, gastroenteritis	<6 mos	6-23 mos	≥2 yrs
A07EA02	hydrocortisone acetate (topical)	mild inflammatory skin conditions	<2 yrs	≥2 yrs	--
C01CA24	epinephrine (auto-injector)	anaphylaxis	--	<15 kg	≥15 kg
C02AC01	clonidine (oral)	attention deficit hyperactivity disorder, hypertension	<1 yr	≥1 yr	--
C07AA06	timolol (eye drops)	ocular hypertension	--	All ages	--
C09AA04	perindopril (oral)	hypertension	All ages	--	--
C09AA05	ramipril (oral)	hypertension	All ages	--	--
D05AC52	calcipotriol + betamethasone dipropionate (topical)	psoriasis	All ages	--	--
D07AA01	methylprednisolone (topical)	eczema, psoriasis	<4 mos	--	≥4 mos
G03AA05	norethisterone + ethinylestradiol (oral)	contraception, endometriosis, acne, menstrual disorders	All ages	--	--
G03AA07	levonorgestrel + ethinylestradiol (oral)	contraception, acne, menstrual disorders	All ages	--	--
G03AC01	norethisterone (oral)	contraception, endometriosis, menstrual disorders	All ages	--	--
G03AC06	medroxyprogesterone (intravenous)	contraception, menstrual disorders	All ages	--	--
G04BD04	oxybutynin (patch)	incontinence	<12 yrs	≥12 yrs	
H02AB09	hydrocortisone (oral)	inflammation or immunosuppression	--	All ages	--
J01AA08	minocycline (oral)	bacterial infections, acne	<8 yrs	≥8 yrs	--
J01FA06	roxithromycin (oral)	bacterial infections	<6 kg	--	≥6 kg
J01FA10	azithromycin (oral)	bacterial infections, pertussis	--	<1 yr	≥1 yr
M01AB05	diclofenac (oral)	pain, inflammation	<1 yr	≥1 yr	--
M01AC06	meloxicam (oral)	inflammatory pain	All ages	--	--
N02AA05	oxycodone (oral)	pain		<12 yrs	≥12 yrs
N02AJ06	paracetamol/codeine (oral)	pain	<12 yrs	--	≥12 yrs
N03AX11	topiramate (oral)	epilepsy	<2 yrs	--	≥2 yrs

N03AX14	levetiracetam (oral)	epilepsy	<12 yrs	≥12 yrs	--
N05AH03	olanzapine (oral)	schizophrenia, bipolar disorder, behaviour disturbance	<13 yrs	≥13 yrs	--
N05AX08	risperidone (oral)	conduct disorders, behaviour disturbance in autism, schizophrenia, bipolar disorder	<5 yrs	5-14 yrs	≥15 yrs
N06AA02	imipramine (oral)	major depression	All ages	--	--
N06AA09	amitriptyline (oral)	neuropathic pain, migraine	<2 yrs	≥2 yrs	
N06AB03	fluoxetine (oral)	major depression, obsessive-compulsive disorder	<7 yrs	≥7 yrs	--
N06AB04	citalopram (oral)	major depression	<12 yrs	≥12 yrs	--
N06AB10	escitalopram (oral)	major depression	<12 yrs	≥12 yrs	--
N06AX11	mirtazapine (oral)	major depression	All ages	--	--
N06AX16	venlafaxine (oral)	major depression, generalised anxiety disorder, panic disorder	All ages	--	--
N06AX21	duloxetine (oral)	major depression, generalised anxiety disorder, neuropathy	All ages	--	--
N06AX23	desvenlafaxine (oral)	major depression	All ages	--	--
N06BA02	dexamfetamine (oral)	attention deficit hyperactivity disorder	<6 yrs	--	≥6 yrs
N06BA04	methylphenidate (oral)	attention deficit hyperactivity disorder	<6 yrs	--	≥6 yrs
R03AC02	salbutamol (inhaled)	asthma	--	<6 mos	≥6 mos
R03AK06	fluticasone propionate + salmeterol (inhaled)	asthma	<6 yrs	--	≥6 yrs
R03AK07	budesonide + formoterol (inhaled)	asthma	<6 yrs	6-12 yrs	≥12 yrs
R03BA01	beclomethasone (inhaled)	asthma, allergic rhinitis	<5 yrs	--	≥5 yrs
R03BA05	fluticasone propionate (inhaled)	asthma	<1 yr	--	≥1 yr
R03BA08	ciclesonide (inhaled)	asthma, allergic rhinitis	<6 yrs	--	≥6 yrs
R03BB01	ipratropium (inhaled)	asthma	<6 mos	≥6 mos	--
R03CC02	salbutamol (oral liquid)	asthma	All ages	--	
S01AA07	framycetin sulfate (eye drops)	bacterial conjunctivitis	--	All ages	--
S01BA01	dexamethasone (eye drops)	allergic and inflammatory eye conditions	<2 yrs	≥2 yrs	--
S01BA07	fluorometholone (eye drops)	inflammatory eye conditions	<2 yrs	≥2 yrs	--

AMH CDC = Australian Medicines Handbook Children’s Dosing Companion; WHO ATC = World Health Organisation Anatomic Therapeutic Chemical

eTable 7. On- and off-label dispensing rate by World Health Organisation Anatomic Therapeutic Chemical anatomic group and age, 2013-17

	**<1 year**		**1-5 years**		**6-11 years**		**12-17 years**	
	**Dispensing rate per 1000 child-years**	**% of dispensings**	**Dispensing rate per 1000 child-years**	**% of dispensings**	**Dispensing rate per 1000 child-years**	**% of dispensings**	**Dispensing rate per 1000 child-years**	**% of dispensings**
**All medicines**								
On-label	1253.4	84.5	2058.1	95.0	1608.4	91.8	1738.2	78.4
Off-label	230.0	15.5	107.5	5.0	142.8	8.2	478.5	21.6
**A – Alimentary tract**								
A01A – Stomatological preparations								
On-label	15.4	100.0	3.2	99.9	1.0	97.4	1.5	94.0
Off-label	0.0	0.0	<0.1	0.1	<0.1	2.6	0.1	6.0
A02B – Drugs for peptic ulcer and gastro-oesophageal reflux disease								
On-label	1.3	1.5	9.8	76.6	15.4	83.5	35.0	82.4
Off-label	82.0	98.5	3.0	23.4	3.1	16.5	7.5	17.6
A03F – Propulsives								
On-label	0.4	100.0	0.4	66.1	1.5	79.4	12.8	88.6
Off-label	0.0	0.0	0.2	33.9	0.4	88.6	1.6	11.4
A04A – Antiemetics and antinauseants								
On-label	3.8	100.0	11.8	81.8	11.1	99.8	11.7	97.4
Off-label	0.0	0.0	2.6	18.2	<0.1	0.2	0.3	2.6
A06A – Drugs for constipation								
On-label	2.3	78.3	5.4	91.9	8.7	99.8	7.1	97.0
Off-label	0.6	21.7	0.5	8.1	<0.1	0.2	0.2	3.0
A10A – Insulins and analogues								
On-label	<0.1	38.5	1.1	85.2	5.5	96.4	15.5	95.3
Off-label	<0.1	61.5	0.2	14.8	0.2	3.6	0.8	4.7
**B – Blood**								
B03A – Iron preparations								
On-label	3.4	99.6	5.1	99.3	2.5	97.6	3.8	71.0
Off-label	<0.1	0.4	<0.1	0.7	0.1	2.4	1.5	29.0
**C – Cardiovascular System**								
C01C – Cardiac stimulants excluding cardiac glycosides								
On-label	2.7	100.0	10.0	86.5	15.2	99.9	10.3	99.9
Off-label	0.0	0.0	1.5	13.5	<0.1	0.1	<0.1	0.1
C02A – Antiadrenergic agents, centrally acting								
On-label	0.0	0.0	0.0	0.0	0.0	0.0	0.0	0.0
Off-label	0.1	100.0	3.1	100.0	23.0	100.0	18.6	100.0

	<1 year		1-5 years		6-11 years		12-17 years	
	**Dispensing rate per 1000 child-years**	**% of dispensings**	**Dispensing rate per 1000 child-years**	**% of dispensings**	**Dispensing rate per 1000 child-years**	**% of dispensings**	**Dispensing rate per 1000 child-years**	**% of dispensings**
C07A – Beta blocking agents								
On-label	0.8	55.8	0.3	27.2	0.9	42.0	4.4	58.8
Off-label	0.6	44.2	0.7	72.8	1.3	58.0	3.1	41.2
**D – Dermatologicals**								
D06B – Chemotherapeutics for topical use								
On-label	1.1	100.0	1.7	100.0	1.3	100.0	1.8	100.0
Off-label	0.0	0.0	0.0	0.0	0.0	0.0	0.0	0.0
D07A – Corticosteroids, plain								
On-label	109.7	57.2	106.9	78.4	67.4	85.5	68.6	89.3
Off-label	82.0	42.8	29.4	21.6	11.4	14.5	8.2	19.4
D10A – Anti-acne preparations for topical use								
On-label	0.0	0.0	0.0	0.0	0.0	0.0	40.1	100.0
Off-label	<0.1	100.0	0.1	100.0	1.0	100.0	0.0	0.0
D10B – Anti-acne preparations for systemic use								
On-label	0.0	0.0	0.0	0.0	0.0	0.0	41.1	100.0
Off-label	<0.1	100.0	<0.1	100.0	0.2	100.0	0.0	0.0
D11A – Other dermatological preparations								
On-label	109.7	57.2	106.9	78.4	67.4	85.5	68.6	89.3
Off-label	82.0	42.8	29.4	21.6	11.4	14.5	8.2	10.7
**G – Genitourinary And Sex Hormones**								
G03A – Hormonal contraceptives for systemic use								
On-label	0.0	13.2	0.0	4.4	0.0	4.0	9.6	7.6
Off-label	0.1	86.8	0.2	95.6	0.5	96.0	115.8	92.4
**H – Systemic Hormones**								
H01B – Posterior pituitary lobe hormones								
On-label	0.1	100.0	0.2	100.0	0.7	99.8	0.6	98.2
Off-label	0.0	0.0	0.0	0.0	0.0	0.2	0.0	1.8
H02A – Corticosteroids for systemic use, plain								
On-label	160.6	99.7	240.6	99.2	104.5	97.6	43.4	92.0
Off-label	0.5	0.3	2.0	0.8	2.5	2.4	3.8	8.0
H03A – Thyroid preparations								
On-label	1.0	98.7	1.2	99.9	1.9	100.0	4.3	99.7
Off-label	0.0	1.3	0.0	0.1	0.0	0.0	0.0	0.3

	<1 year		1-5 years		6-11 years		12-17 years	
	**Dispensing rate per 1000 child-years**	**% of dispensings**	**Dispensing rate per 1000 child-years**	**% of dispensings**	**Dispensing rate per 1000 child-years**	**% of dispensings**	**Dispensing rate per 1000 child-years**	**% of dispensings**
H04A – Glycogenolytic hormones								
On-label	0.1	100.0	0.5	100.0	1.7	100.0	3.2	100.0
Off-label	0.0	0.0	0.0	0.0	0.0	0.0	0.0	0.0
**J – Systemic Antiinfectives**								
J01A – Tetracyclines								
On-label	0.0	0.0	0.0	0.0	1.9	80.0	119.7	72.7
Off-label	0.1	100.0	0.2	100.0	0.5	20.0	45	27.3
J01C – Beta-lactam antibacterials, penicillins								
On-label	573	100.0	843.1	100.0	436.9	99.8	335.4	100
Off-label	0.0	0.0	0.0	0.0	1.0	0.2	0.1	0.0
J01D – Other beta-lactam antibacterials								
On-label	129	100.0	275.4	100.0	188.1	100.0	164.8	100
Off-label	0.0	0.0	0.0	0.0	0.0	0.0	0.0	0.0
J01E – Sulfonamides and trimethoprim								
On-label	23.7	99.9	35.2	99.7	20.9	100.0	23.5	100.0
Off-label	0.0	0.1	0.1	0.3	0.0	0.0	0.0	0.0
J01F – Macolides, lindosamines and streptogramins								
On-label	48.8	84.2	118.6	100.0	80.8	100.0	99.3	100.0
Off-label	9.2	15.8	0.0	0.0	0.0	0.0	0.0	0.0
J01X – Other antibacterials								
On-label	2.9	100.0	9.4	99.8	9.3	99.6	12.2	98.9
Off-label	0.0	100.0	0.0	0.2	0.0	0.4	0.1	1.1
J05A – Direct acting antivirals								
On-label	0.0	25.0	0.2	74.8	0.5	44.9	3.2	95.2
Off-label	0.1	75.0	0.1	25.2	0.6	55.1	0.2	4.8
**M – Musculoskeletal**								
M01A – Antiinflammatory and antirheumatic products, non-steroids								
On-label	0.0	23.3	0.1	14.1	1.1	41.4	21.1	59.9
Off-label	0.1	76.7	0.7	85.9	1.6	58.6	14.1	40.1
**N – Nervous System**								
N02A – Opioids								
On-label	0.1	12.1	0.2	6.7	0.1	3.0	48	94.5
Off-label	0.5	87.9	3.2	93.3	4.3	97	2.8	5.5

	<1 year		1-5 years		6-11 years		12-17 years	
	**Dispensing rate per 1000 child-years**	**% of dispensings**	**Dispensing rate per 1000 child-years**	**% of dispensings**	**Dispensing rate per 1000 child-years**	**% of dispensings**	**Dispensing rate per 1000 child-years**	**% of dispensings**
N02B – Other analgesics and antipyretics								
On-label	6.5	99.9	8.0	99.3	6.8	96.1	4.8	61.7
Off-label	0.0	0.1	0.1	0.7	0.3	3.9	3.0	38.3
N02C – Antimigraine preparations								
On-label	0.0	0.0	0.3	67	0.9	48.1	2.0	18.3
Off-label	0.0	100.0	0.1	33	1.0	51.9	8.8	81.7
N03A – Antiepileptics								
On-label	1.2	29.2	14.2	78.9	34	85.7	53.4	98.4
Off-label	2.9	70.8	3.8	21.1	5.7	14.3	0.9	1.6
N05A – Antipsychotics								
On-label	0.0	0.0	0.0	0.1	0.1	0.4	18	44.1
Off-label	0.0	100.0	2.0	99.9	20.4	99.6	22.8	55.9
N05B – Anxiolytics								
On-label	0.1	72.2	0.4	95.3	1.1	94.7	5.2	87.7
Off-label	0.0	27.8	0.0	4.7	0.1	5.3	0.7	12.3
N06A – Antidepressants								
On-label	0	0.0	0	0.0	9.2	25.6	63.3	28.2
Off-label	0.2	100.0	2.1	100.0	26.9	74.4	161.6	71.8
N06B – Psychostimulants, agents used for ADHD and nootropics								
On-label	0.0	0.0	0.0	0.0	181.3	100.0	166.8	99.9
Off-label	0.0	0.0	4.9	100.0	0.0	0.0	0.1	0.1
**P – Antiparasitics**								
P03A – Ectoparasiticides, including scabicides								
On-label	2.4	70.2	4.4	100.0	4.5	100.0	5.5	100.0
Off-label	1.0	29.8	0.0	0.0	0.0	0.0	0.0	0.0
**R – Respiratory**								
R03A – Adrenergics, inhalants								
On-label	30.9	84.0	106.3	88.2	145.4	96.6	142.3	99.3
Off-label	5.9	16	14.2	11.8	5.2	3.4	1.1	0.7
R03B – Other drugs for obstructive airway diseases, inhalants								
On-label	1.5	17.2	86.1	96.3	81.5	97.6	27.0	94.4
Off-label	7.2	82.8	3.3	3.7	2.0	2.4	1.6	5.6
R03C – Adrenergics for systemic use								
On-label	0.0	0.0	0.0	0.0	0.0	0.0	0.0	0.0
Off-label	5.8	100.0	6.8	100.0	1.8	100.0	0.5	100.0

	<1 year		1-5 years		6-11 years	
	**Dispensing rate per 1000 child-years**	**% of dispensings**	**Dispensing rate per 1000 child-years**	**% of dispensings**	**Dispensing rate per 1000 child-years**	**% of dispensings**
R03D – Other systemic drugs for obstructive airway disease						
On-label	0.0	0.0	47.8	98.5	61.6	100.0	16.5	100.0
Off-label	0.2	100.0	0.7	1.5	0.0	0.0	0.0	0.0
**S – Sensory Organs**						
S01A – Ophthalmological antiinfectives						
On-label	0.0	0.0	47.8	98.5	61.6	100.0	16.5	100.0
Off-label	0.2	100.0	0.7	1.5	0.0	0.0	0.0	0.0
S01B – Ophthalmological antiinflammatory agents						
On-label	0.0	0.0	0.0	0.0	0.0	0.0	0.0	0.0
Off-label	2.9	100.0	4.2	100.0	6.9	100.0	6.9	100
S02A – Otological antiinfectives						
On-label	1.5	88	3.9	95.5	1.9	93.6	0.6	90.8
Off-label	0.2	12.0	0.2	4.5	0.1	6.4	0.1	9.2
S02C – Otological corticosteroids and antiinfectives in combination						
On-label	23.5	100.0	37.6	100.0	42.1	100.0	29.6	100.0
Off-label	0.0	0.0	0.0	0.0	0.0	0.0	0.0	0.0
S03A – Ophthalmological and otological antiinfectives						
On-label	0.0	0.0	0.0	0.0	0.0	0.0	0.0	0.0
Off-label	10.4	100.0	6.0	100.0	2.4	100.0	1.5	100.0

Supplemental Figures

eFigure 1a: Average yearly prevalence per 1000 children by age, sex and World Health Organisation Anatomic Therapeutic Chemical classification pharmacological subgroup, 2013-17

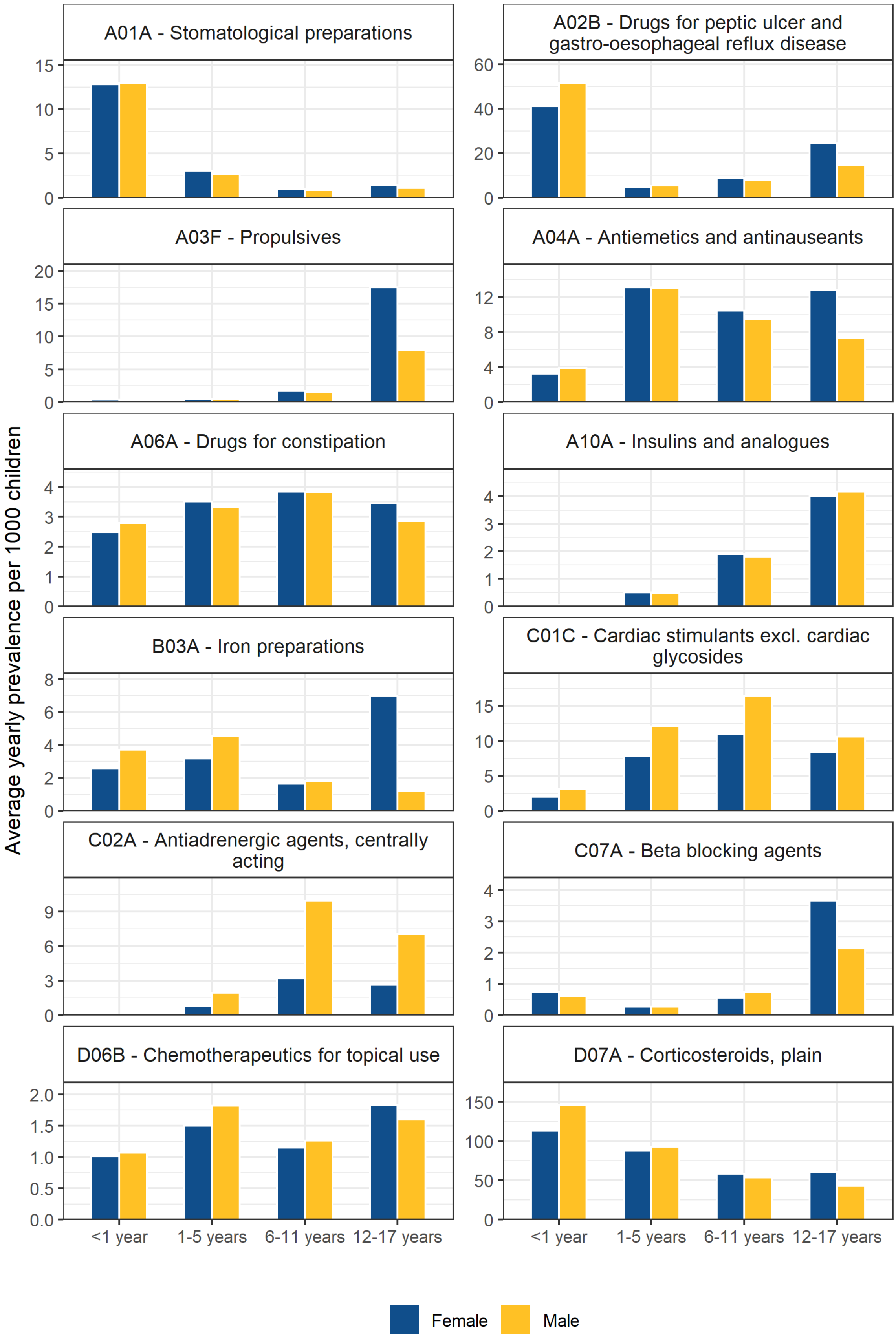

eFigure 1b. Average yearly prevalence per 1000 children by age, sex and World Health Organisation Anatomic Therapeutic Chemical classification pharmacological subgroup, 2013-17

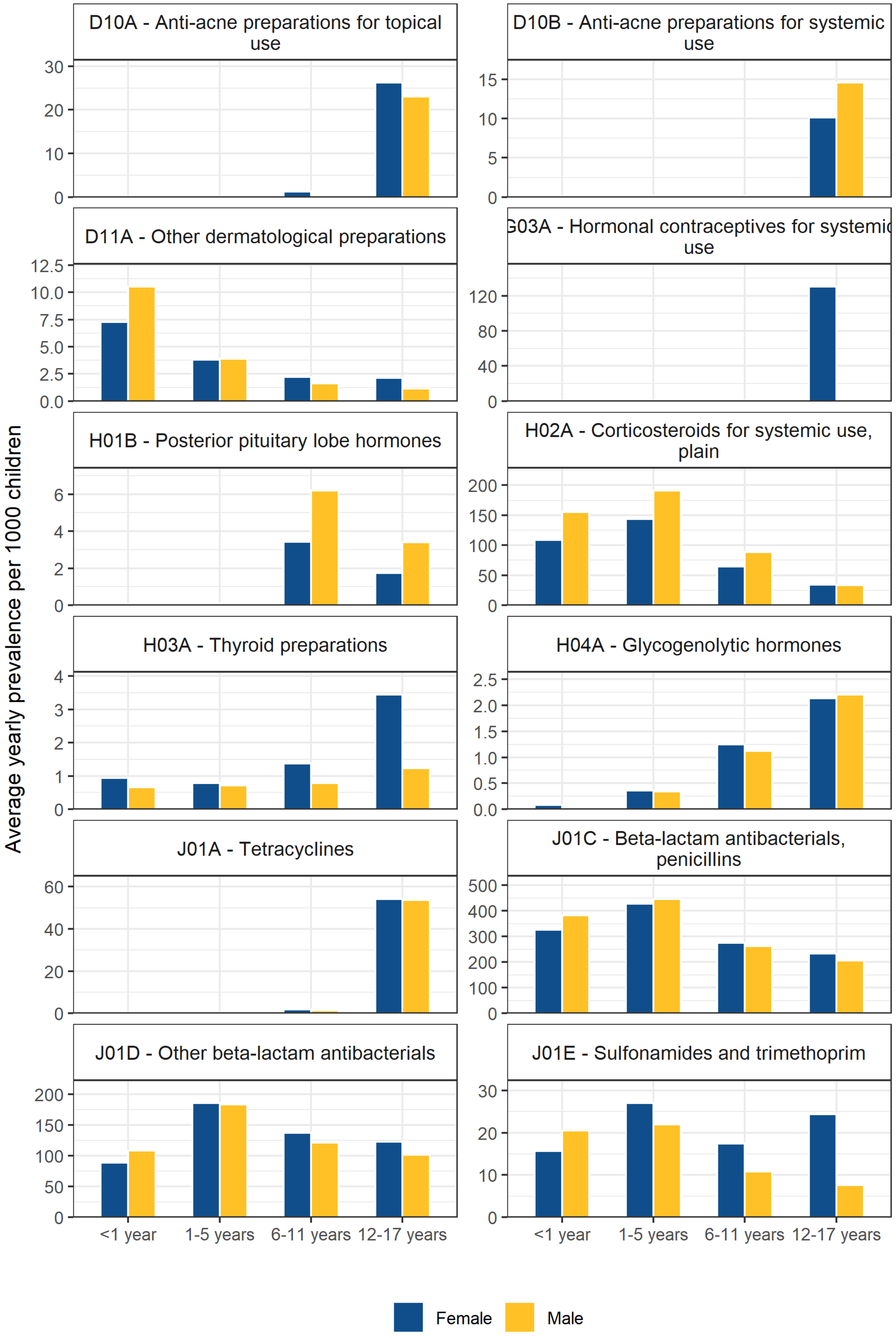

eFigure 1c. Average yearly prevalence per 1000 children by age, sex and World Health Organisation Anatomic Therapeutic Chemical classification pharmacological subgroup, 2013-17

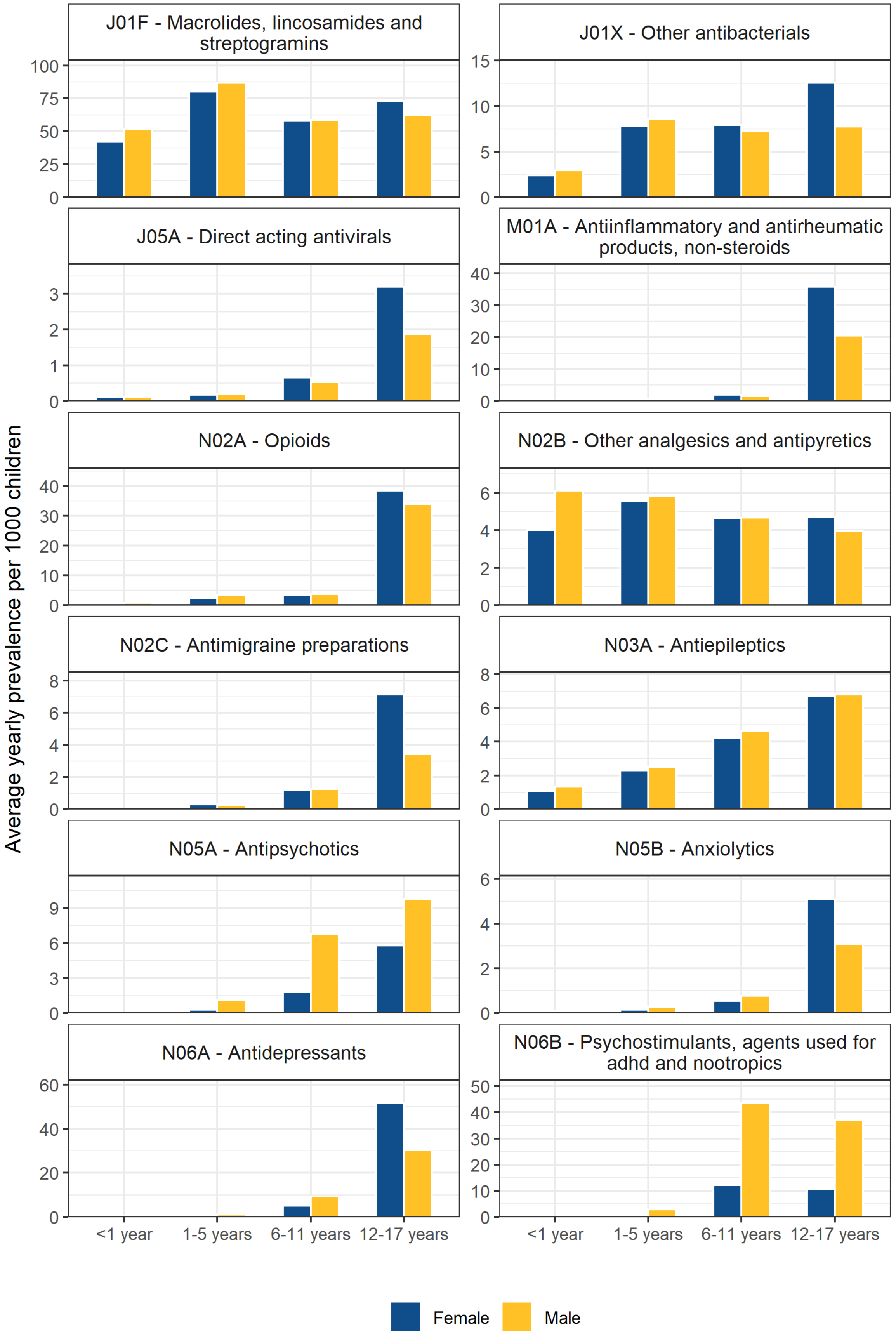

eFigure 1d. Average yearly prevalence per 1000 children by age, sex and World Health Organisation Anatomic Therapeutic Chemical classification pharmacological subgroup, 2013-17

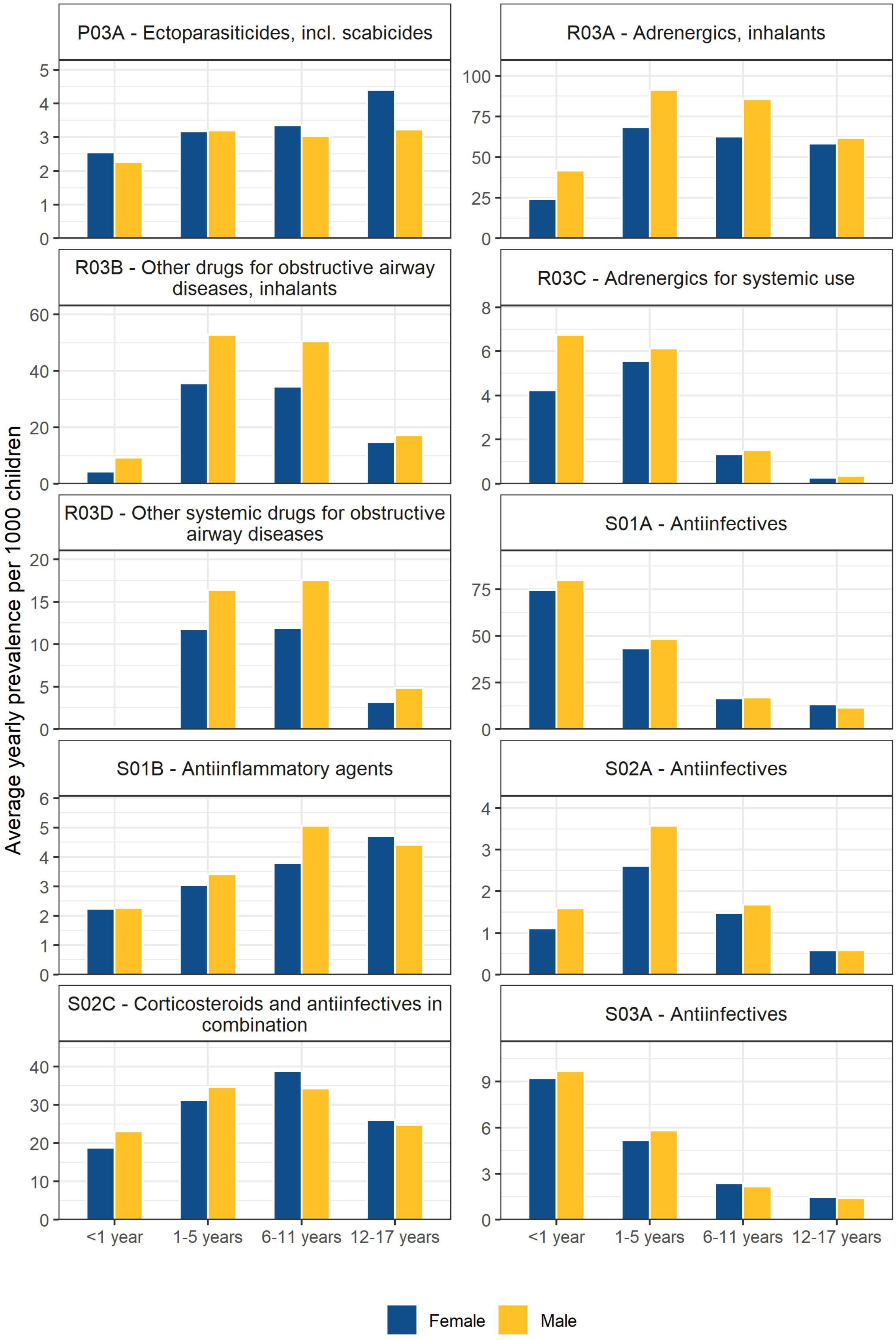

Supplement: Supplementary file 1 — Supplementary Material [file PPE-36-726-s001.pdf]
